# Supplementary material for: Genome-wide identification and transcriptional expression analysis of superoxide dismutase (SOD) family in wheat (Triticum aestivum)
Source: PeerJ. 2019 Nov 19;7:e8062. doi: 10.7717/peerj.8062 (PMC6873880; doi:10.7717/peerj.8062)
Supplement: File S1 [file peerj-07-8062-s006.docx]

Supplemental File 1

**The gene sequences used in this research.**

Including CDS and protein sequences of TaSODs, and protein sequences of other plant SOD gens.

1. **CDS sequences**

>TaSOD1.1.a

CAGATCACACACACAATGGTGAAGGCTGTGGCTGTGCTTACCGGCAGTGAGGGTGTCAAGGGCACCATCTTCTTCACCCAGGAGGGAGAGGGCCCGACCACCGTGACGGGAAGTGTCACCGGACTCAAGGAAGGGCTCCACGGCTTCCATGTGCACGCTCTTGGCGACACCACCAATGGCTGCATGTCAACTGGACCACACTTCAACCCTGCTGGTCATGTGCATGGGGCACCCGAAGATGAAATCCGCCATGCTGGTGATCTTGGAAATGTGACAGCTGGAGTGGATGGTGTTGCTAGCATCAATATTACTGACTGCCATATCCCCCTTACTGGACCAAATTCAATTGTTGGCCGTGCTGTTGTCGTCCATGGTGACGCTGATGATCTTGGCAAGGGTGGACATGAGCTGAGCAAGAGCACTGGAAACGCTGGTGCACGTGTTGCTTGCGGAATCATCGGGCTCCAGGGCTAA

>TaSOD1.1b

ATGGTGAAGGCTGTGGCTGTGCTTACCGGCAGTGAGGGTGTCAAGGGCACCATCTTCTTCACCCAGGAGGGAGAGGGCCCGACCACCGTGACGGGAAGTGTCACCGGACTCAAGGAAGGGCTCCACGGCTTCCATGTGCACGCTCTTGGCGACACCACCAATGGCTGCATGTCAACTGGACCACACTTCAACCCTGCTGGTCATGTGCATGGGGCACCCGAAGATGAAATCCGCCATGCTGGTGTTGCTAGCATCAATATTACTGACTGCCATATCCCCCTTACTGGACCAAATTCAATTGTTGGCCGTGCTGTTGTCGTCCATGGTGACGCTGATGATCTTGGCAAGGGTGGACATGAGCTGAGCAAGAGCACTGGAAACGCTGGTGCACGTGTTGCTTGCGGAATCATCGGGCTCCAGGGCTAA

>TaSOD1.2

ATGGTGGGCTTCCTCCGGGCCTTCACCGCCGCATCCGCCGTGCCAGCAGCAGCCGTCGCCGCGGCCGCCCTCTCCTCCTCCTCCTCCTCCCCCTCCCGCTCCCCCTCCTCCAGGCTCCGCTTCCCCCTGCCTCCCTCCCTCTCCGCGTTCGCCGCCTCCTCGTCCTCCTCTGCCCCCGTGCGCGCGCCGACCGCCGCCCCTCCGATGGCCGCCGCCGCCACCGCCGATCTCTCTGCCCCCGACAAGGGTACCGCGCTTCCGGAGCTCACGACCGAATTCATGGTGGACATGAAATGTGAGGGCTGTGTCACAGCAGTGAAAAACAGGCTTCAAACACTTGAAGGAATACAAAACATCGAAGTGGATTTGAATAACCAGGTAGTTAGAGTTCGTGGGTCTCTCCCAGTGAAGATAATGTTGGATGCTCTGCATCAAACAGGACGAGATGCACGTCTGATTGGGCAAGGGAACCCAGATGATTTCCTAGTTTCTGCTGCCGTGGCTGAGTTCAAAGGGCCAGTTATATTTGGTGTTGTTCGTCTAGCCCAAGTTAACATGGAATTGGCTAGAGTTGAGGCTACTTTTAGTGGTCTATCACCTGGTAAACATGGATGGTCAATAAATGAATTTGGGGATTTGACAAAAGGTGCAGAAAGTACTGGCAAAGTATACAATCCACAGGATTATCTGTCTGATAAGCCCCTTGGTGACCTGGGAACACTAGAAGCTGGAGAGAATGGAGAAGCCCAGTTTTCAGGATCAAAAGAGAAACTGAAAGTTGTCGACTTGATCGGTCGCTCTATTGCGCTGTATGCAACCGAGGACAGATCAGACCCAGGCATTGCAGCTGCCGTTGTCGCGAGAAGTGCGGGTGTTGGGGAGAACTACAAGAAACTCTGCACATGCGATGGTGTCACCATTTGGGAATCGAGCTAA

>TaSOD1.3

ATGGTGGGCTTCCTCCGGGCCTTCACCGCCGCATCCGCCGTGCCAGCAGCAGCCGTCGCCGCGGTCGCCCTCTCCTCCTCCTCCCCCTCTCGCTCCCCCTCTTCCAGGCTCCGCTTCCCCCTGCCTCCCTCCCTCTCCGCGTTCGCCGCCTCCTCGGCCTCCTCTTCCCCCGTGCGCGCGCCGACCGCCGCCCCTCCGATGGCCGCCGCCGCCACTGCCGATCTCTCTGCTCCCGACAAGGGCACCGCGCTTCCGGAGCTCACGACCGAATTCATGGTGGACATGAAATGTGAGGGCTGTGTCACAGCAGTGAAAAATAAGCTTCAAACACTTGAAGGAATACAGAACATCGAAGTGGATTTCAATAACCAGGTAGTTAGAGTTCGTGGGTCTCTCCCAGTGAAGCTAATGTTGGATGCTCTGCGTCAAACAGGACGAGATGCACGTCTGATTGGGCAAGGGAACCCAGATGATTTCCTAGTTTCTGCTGCCGTGGCTGAGTTCAAAGGGCCAGTTATATTTGGTGTTGTTCGTCTAGCCCAAGTTAACATGGAATTGGCTAGAGTTGAGGCTACTTTTAGTGGTCTATCACCTGGTAAACATGGATGGTCAATAAATGAATTTGGGGATTTGACAAAAGGTGCAGAAAGTACTGGCAAAGTATACAATCCACAGGATTATCTATCTGATAAGCTCCTTGGTGACCTGGGAACACTAGAAGCTGGAGAAAGAGAAGCCCAGTTTTCAGGATCAAAAGAGAAACTGAAAGTTGTCGACTTGATCGGTCGCTCTATTGCGCTGTATGCAACCGAGGACAGATCAGACGCGGGCCTTGCAGCTGCCGTTGTCGCGAGAAGTGCGGGTGTTGGGGAGAACTACAAGAAACTCTGCACATGCGACGGTGTCACCATTTGGGAATCGAGCTAA

>TaSOD1.4

ATGGTGAAGGCTGTGGCTGTGCTTACCGGCAGTGAGGGTGTCAAGGGCACCATCTTCTTCACCCAGGAGGGAGATGGCCCGACCACCGTGACGGGAAGCGTCACTGGACTCAAGGAAGGGCTCCACGGCTTCCACGTGCACGCTCTTGGTGACACCACCAACGGCTGCATGTCAACTGGACCACACTTCAACCCTGCTGGTCATGTGCATGGGGCACCAGAAGATGAAATCCGCCATGCTGGTGATCTTGGAAATGTGACAGCTGGAGCGGATGGTGTTGCTAACATCAATGTTACTGACTGCCATATCCCCCTTACTGGACCAAATTCAATTGTTGGCCGTGCTGTTGTCGTCCACGGTGACGCTGATGATCTTGGCAAGGGTGGACATGAGCTTAGCAAGAGCACTGGAAACGCTGGTGCGCGTGTTGCTTGCGGAATCATCGGGCTCCAGGGCTAA

>TaSOD1.5a

ATGGTGGGCTTCCTCCGGGCCTTCACCGCCGCATCCGCCGTGCCAGCAGCAGCCGTCGCCGCGGCCGCCCTCTGCTCCTCCTCCTCCCCCTCCCGCTCCCCCTCCTCCAGGCTCCGCTTCCCCCTGCCCTCCCTCTCCGCGTTCGCCGCCTCCTCGTCCTCCTCTTCTCCCGTGCGCGCGCCGACCGCCGCCCCTCCGATGGCCGCCGCCGCCACAGCCGATCTCTCTGCCCCCGACAAGGGCACCGCGCTTCCGGAGCTCACGACCGAATTCATGGTGGACATGAAATGTGAGGGCTGTGTCACAGCAGTGAAAAACAGGCTTCAAACACTTGAAGGAATACAAAACATCGAAGTGGATTTGAATAACCAAGTAGTTAGAGTTCGTGGGTCTCTCCCAGTGAAGATAATGTTGGATGCTCTGCATCAAACAGGACGAGATGCACGTCTGATTGGGCAAGGGAACCCAGATGATTTCCTAGTTTCTGCTGCCGTGGCTGAGTTCAAAGGGCCAGTTATATTTGGTGTTGTTCGTCTAGCCCAAGTTAACATGGAATTGGC

TAGAATTGAGGCTACTTTTAGTGGTCTATCACCTGGTAAACATGGATGGTCAATAAATGAATTTGGGGATTTGACAAAAGGTGCAGAAAGTACTGGCAAAGTATACAATCCACAGGATTATCTATCTGATAAGCCCCTTGGTGACCTGGGAACACTAGAAGCTGGAGAAAACGGAGAAGCCCAGTTTTCAGGATCAAAAGAGAAACTGAAAGTTGTCGACTTGATCGGTCGCTCTATTGCGCTGTATGCAACCGAGGACAGATCAGACCCAGGCATTGCAGCTGCCGTGGTCGCGAGAAGTGCGGGCGTTGGGGAGAACTACAAGAAACTCTGCACATGCGATGGTGTCACAATTTGGGAATCGAGCTAA

>TaSOD1.5b

ATGGTGGGCTTCCTCCGGGCCTTCACCGCCGCATCCGCCGTGCCAGCAGCAGCCGTCGCCGCGGCCGCCCTCTGCTCCTCCTCCTCCCCCTCCCGCTCCCCCTCCTCCAGGCTCCGCTTCCCCCTGCCCTCCCTCTCCGCGTTCGCCGCCTCCTCGTCCTCCTCTTCTCCCGTGCGCGCGCCGACCGCCGCCCCTCCGATGGCCGCCGCCGCCACAGCCGATCTCTCTGCCCCCGACAAGACCGAATTCATGGTGGACATGAAATGTGAGGGCTGTGTCACAGCAGTGAAAAACAGGCTTCAAACACTTGAAGGAATACAAAACATCGAAGTGGATTTGAATAACCAAGTAGTTAGAGTTCGTGGGTCTCTCCCAGTGAAGATAATGTTGGATGCTCTGCATCAAACAGGACGAGATGCACGTCTGATTGGGCAAGGGAACCCAGATGATTTCCTAGTTTCTGCTGCCGTGGCTGAGTTCAAAGGGCCAGTTATATTTGGTGTTGTTCGTCTAGCCCAAGTTAACATGGAATTGGCTAGAATTGAGGCTACTTTTAGTGGTCTATCACCTGGTAAACATGGATGGTCAATAAATGAATTTGGGGATTTGACAAAAAGGTGCAGAAAGTACTGGCAAAGTATACAATCCACAGGATTATCTATCTGATAAGCCCCTTGGTGACCTGGGAACACTAGAAGCTGGAGAAAACGGAGAAGCCCAGTTTTCAGGATCAAAAGAGAAACTGAAAGTTGTCGACTTGATCGGTCGCTCTATTGCGCTGTATGCAACCGAGGACAGATCAGACCCAGGCATTGCAGCTGCCGTGGTCGCGAGAAGTGCGGGCGTTGGGGAGAACTACAAGAAACTCTGCACATGCGATGGTGTCACAATTTGGGAATCGAGCTAA

>TaSOD1.6a

ATGGCAGGGAAACCCGGCAGCCTCAAGGGTGTCGCCCTCATCAGCGGCGGTGGCGCCGACAGCGCTGTCGCCGGCGCCCTCCACTTCGTCCAAGACCCCTCCTCCGGGTATACCGAGGTGAGGGGGAGGGTCTCGGGCCTCGCCCCGGGCCTCCACGGCTTCCACATCCACGCCTTCGGCGACACCACCAACGGCTGCAACTCCACCGGACCCCATTTCAATCCTCATAATAAGTCCCATGGAGCACCGGTTGATGATGAACGACATGTGGGCGACCTGGGAAACATACAAGCCAACAAGGATGGTGTTGCAGAAATCTTCATAAAGGACTTGCAGATTTCACTAAGGGGGCCTCATTCCATACTGGGAAGGGCAGTTGTCGTTCATGCTGATTCTGATGACCTAGGAAAGGGTGGCCATGAACTCAGCAAGTCAACAGGAAATGCAGGAGCCAGAATTGGATGTGGTATCATTGGAATTCAGCCGGCTGTTTAA

>TaSOD1.6b

ATGGCAGGGAAACCCGGCAGCCTCAAGGGTGTCGCCCTCATCAGCGGCGGTGGCGCCGACAGCGCTGTCGCCGGCGCCCTCCACTTCGTCCAAGACCCCTCCTCCGGGTATACCGAGGTGAGGGGGAGGGTCTCGGGCCTCGCCCCGGGCCTCCACGGCTTCCACATCCACGCCTTCGGCGACACCACCAACGGCTGCAACTCCACCGGACCCCATTTCAATCCTCATAATAAGTCCCATGGAGCACCGGTTGATGATGAACGACATGTGGGCGACCTGGGAAACATACAAGCCAACAAGGATGGTGTTGCAGAAATCTTCATAAAGGACTTGCAGATTTCACTAAGGGGGCCTCATTCCATACTGGGAAGGGCAGTTGTCGTTCATGCTGATTCTGATGACCTAGGAAAGGGTGGCCATGAACTCAGCAAGTCAACAGGAAATGCAGGAGCCAGAATTGGATGTGGTAAATGGCAAACCCTTTATTTGTTTTCAACACTGAGCAAATGCACACACAAGGTATCATTGGAATTCAGCCGGCTGTTTAACAAAAAAAATACAAGAGCCGTTGGAGTCCATGTTGGTATTGCTGCGCGCAGACAAGGAAACAGTGATATCGTTGAAGATCATACATTTTGA

>TaSOD1.7a

ATGGCAGGGAAACCCGGCAGCCTCAAGGGCGTCGCCCTCATCAGCGGCGGTAGCGCCGACAGCGCTGTCGCCGGCGCCCTCCACTTCGTCGAAGACCCCTCCTCCGGGTATACCGAGGTGAGGGGGAGGGTCTCGGGCCTCGCCCCGGGCCTCCACGGCTTCCACATCCACGCCTTCGGCGACACCACCAACGGCTGCAACTCCACCGGACCCCATTTCAATCCTCATAATAAGTTCCATGGAGCACCGATGGATGATGAACGACATGTGGGCGACCTGGGAAACATACAAGCCAACAAGGATGGTGTTGCAGAAATCTTCATAAAGGACTTGCAGATTTCACTAAGGGGGCCTCATTCCATATTGGGAAGGGCAGTTGTTGTTCATGCTGATTCTGATGACCTGGGAAAGGGTGGCCATGAACTCAGCAAGTCAACAGGAAATGCAGGAGCCAGAATTGGATGTGGTGTAATTGGAATTCAACCTGCTGTTTAA

>TaSOD1.7b

ATGGCAGGGAAACCCGGCAGCCTCAAGGGCGTCGCCCTCATCAGCGGCGGTAGCGCCGACAGCGCTGTCG

CCGGCGCCCTCCACTTCGTCGAAGACCCCTCCTCCGGGTATACCGAGGTGAGGGGGAGGGTCTCGGGCCTCGCCCCGGGCCTCCACGGCTTCCACATCCACGCCTTCGGCGACACCACCAACGGCTGCAACTCCACCGGACCCCATTTCAATCCTCATAATAAGTTCCATGGAGCACCGATGGATGATGAACGACATGTGGGCGACCTGGGAAACATACAAGCCAACAAGGATGGTGTTGCAGAAATCTTCATAAAGGACTTGCAGATTTCACTAAGGGGGCCTCATTCCATATTGGGAAGGGCAGTTGTTGTTCATGCTGATTCTGATGACCTGGGAAAGGGTGGCCATGAACTCAGCAAGTCAACAGGAAATGCAGGAGCCAGAATTGGATGTGGTAAATGGCAAACCCTTCATTTGTTTTCAACACTGAGCAAATGCACGCACAAGGTGTAA

>TaSOD1.8a

ATGGCAGGGAAACCCGTTAGCCTCAAGGGCGTCGCCCTCATCAGCGGCGGTGCCGCCGACAGCGCTGTCGCCGGCGCCCTCCACTTCGTCCAAGACCCCTCCTCCGGGTATACCGAGGTGAGGGGGTGGGTCTCGGGCCTCGCCCCGGGCCTCCACGGCTTCCACATCCACGCCTTCGGCGACACCACCAACGGCTGCAACTCCACCGGACCCCATTTCAATCCTCATAATAAGTCCCATGGAGCACCGGTTGATGACGAACGACATGTGGGCGACCTGGGAAACATACAAGCCAACAAGGATGGTGTTGCAGAAATCTTCATAAAGGACTTGCAGATTTCACTAAGGGGGCCTCATTCCATATTGGGAAGGGCAGTTGTTGTTCATGCTGATTCTGATGACCTGGGAAAGGGACCATGGGTGACACTGGATCATGACTAA

>TaSOD1.8b

ATGGCAGGGAAACCCGTTAGCCTCAAGGGCGTCGCCCTCATCAGCGGCGGTGCCGCCGACAGCGCTGTCGCCGGCGCCCTCCACTTCGTCCAAGACCCCTCCTCCGGGTATACCGAGGTGAGGGGGTGGGTCTCGGGCCTCGCCCCGGGCCTCCACGGCTTCCACATCCACGCCTTCGGCGACACCACCAACGGCTGCAACTCCACCGGACCCCATTTCAATCCTCATAATAAGTCCCATGGAGCACCGGTTGATGACGAACGACATGTGGGCGACCTGGGAAACATACAAGCCAACAAGGATGGTGTTGCAGAAATCTTCATAAAGGACTTGCAGATTTCACTAAGGGGGCCTCATTCCATATTGGGAAGGGCAGTTGTTGTTCATGCTGATTCTGATGACCTGGGAAAGGGTGGCCATGAACTCAGTAAATCAACAGGAAATGCAGGAGCCAGAATTGGATGTGGTATCATTGGAATTCAGCCTGCTGTTTAA

>TaSOD1.9

ATGGCCGCTCAGAGCCTCCTCTTTGCCGCCGCCGCGCCTCTCTTCCAGGCTCCTGCCTCTGCCCGCCCTT

TCCAGTCGCTCCGAATTGTCTGCACCCCAGAAGGCGCCACCGCCGCCGCCAGGGCGCTCGTCGTCGCCGA

CGCCACCAAGAAGGCAGTCGCGGTGCTCAAGGGCACCTCGCAGGTCGAGGGCGTCGTCACGCTCACCCAGGAAGACGACGGTCCTACGACGGTGAACGTTCGTATCACTGGACTTGCTCCTGGACTTCATGGCTTCCACCTCCATGAGTTTGGTGACACGACTAATGGATGCATATCAACAGGTCCACATTTTAACCCAAACGGCCTGACACATGGTGCACCAGAAGATGAAGTCCGTCATGCGGGTGACCTGGGAAACATTGTTGCCAATGCTGAAGGTGTGGCGGAGACAACCATTGTCGATAGCCAGATTCCTTTGACTGGCCCTAATGCAGTTGTTGGGAGAGCGTTTGTTGTTCATGAGCTTGAAGATGACTTGGGAAAAGGTGGGCATGAGCTCAGCCTCAGTACTGGAAATGCTGGTGGAAGACTTGCATGTGGTGTTGTTGGCCTGACCCCGTTGTAG

>TaSOD1.10

ATGGCCGCGCAGAGCCTCCTCTTTGCCGCCGCCGCGCCTCTCTTCCAGGTTCCTGCCTCTGCCCGCCCTTTCCAGTCGCTCCGAATTGTCTCCACTCCAGGAGGCGCCACCGCCGCCGCCAGGGCGCTCGTCGTCGCCGACGCCACCAAGAAGGCAGTCGCGGTGCTCAAGGGCACCTCCCAGGTCGAGGGCGTCGTCACGCTCACCCAGGAAGACGACGGTCCCACGACGGTGAACGTTCGTATCACTGGACTTGCTCCTGGACTTCATGGCTTCCACCTCCATGAGTTCGGTGACATGACTAATGGGTGCATATCAACAGGTCCACATTTTAACCCAAACGGCCTGACACATGGTGCACCAGAAGATGAAGTCCGTCATGCGGGTGACCTGGGAAACATTGTTGCCAATGCTGAAGGTGTGGCGGAGACAACCATTGTCGATAGCCAGATTCCTTTGACTGGCCCTAATGCAGTTGTTGGGAGAGCGTTTGTTGTTCATGAGCTTGAAGATGACTTGGGAAAAGGTGGGCATGAGCTCAGCCTCAGTACTGGAAATGCTGGTGGAAGACTTGCATGTGGTGTTGTTGGCCTGACCCCGTTGTAG

>TaSOD1.11a

ATGGCCGCTCAGAGCCTCCTCTTTGCCGCCGCCGCGCCTCTCTTCCAGGCTCCTGCCTCTGCCCGCCCTTTCCAGTCGCTCCGAATTGTCTCCACCCCAGGAGGCGCCACCGCCGCCGCCAGGGCGCTCGTCGTCGCCGACGCCACCAAGAAGGCAGTCGCGGTGCTCAAGGGCTCCTCCCAGGTCGAGGGCGTCGTCACGCTCACCCAGGAAGACGACGGTCCTACGACGGTGAACGTTCGTATCACTGGACTTGCTCCTGGACTTCATGGCTTCCACCTCCATGAGTTTGGTGACACGACTAATGGATGCATATCAACAGGTCCACATTTTAACCCAAACGGCCTGACACATGGTGCACCAGAAGATGAAGTCCGTCATGCGGGTGACCTGGGAAACATTGTTGCCAATGCTGAGGGTGTGGCGGAGACAACCATTGTCGATAGCCAGATTCCTTTGACTGGCCCTAATGCAGTTGTTGGGAGAGCGTTTGTTGTTCATGAGCTTGAAGATGACTTGGGAAAAGGTGGGCATGAGCTCAGCCTCAGTACTGGAAATGCTGGTGGAAGACTTGCATGTGGTGTTGTTGGCCTGACCCCGTTGTAG

>TaSOD1.11b

ATGGCCGCTCAGAGCCTCCTCTTTGCCGCCGCCGCGCCTCTCTTCCAGGCTCCTGCCTCTGCCCGCCCTTTCCAGTCGCTCCGAATTGTCTCCACCCCAGGAGGCGCCACCGCCGCCGCCAGGGCGCTCGTCGTCGCCGACGCCACCAAGAAGGCAGTCGCGGTGCTCAAGGGCTCCTCCCAGGTCGAGGGCGTCGTCACGCTCACCCAGGAAGACGACGCAGGTCCTACGACGGTGAACGTTCGTATCACTGGACTTGCTCCTGGACTTCATGGCTTCCACCTCCATGAGTTTGGTGACACGACTAATGGATGCATATCAACAGGTCCACATTTTAACCCAAACGGCCTGACACATGGTGCACCAGAAGATGAAGTCCGTCATGCGGGTGACCTGGGAAACATTGTTGCCAATGCTGAGGGTGTGGCGGAGACAACCATTGTCGATAGCCAGATTCCTTTGACTGGCCCTAATGCAGTTGTTGGGAGAGCGTTTGTTGTTCATGAGCTTGAAGATGACTTGGGAAAAGGTGGGCATGAGCTCAGCCTCAGTACTGGAAATGCTGGTGGAAGACTTGCATGTGGTGTTGTTGGCCTGACCCCGTTGTAG

>TaSOD2.1

ATGGCGCTCCGCACATTGGCCGCGAAGAAAACCCTAGGCCTGGCGCTCGGCGGCGCGCGGCCGCTGGCCGCCGCCAGGGGCGTGGCGACGTTCACGCTCCCCGACCTCCCCTACGACTTCGGTGCGCTGGAGCCGGCCGTCTCCGGCGAGATCATGCGCCTGCACCACCAGAAGCACCACGCCACCTACGTCGCCAACTACAACAAGGCGCTCGAGCAGCTCGACGCCGCCGTCAGCAAGGGGGACGCGTCCGCCGTCGTCCACCTCCAGAGCGCCATCAAATTCAACGGCGGCGGTCATGTTAACCATTCAATCTTCTGGAAGAACCTCAAGCCTATTAGCGAGGGTGGTGGTGAGCCACCTCATGGCAAACTTGGCTGGGCCATTGATGAGGATTTTGGTTCTATTGAGAAACTTATAAAGAAGATGAATGCAGAGGGTGCTGCTTTACAAGGATCTGGATGGGTGTGGCTAGCTTTGGATAAAGAGGCCAAAAAGCTTTCAGTTGAAACTACTCCTAATCAGGACCCTCTTGTGACCAAAGGGTCAAACCTGTATCCTTTGTTGGGAATTGATGTCTGGGAGCATGCGTACTACCTGCAGTACAAGAACGTGAGGCCGGACTACCTGACCAACATCTGGAAGGTGGTGAACTGGAAATATGCCGGAGAAGAGTATGAAAAAGTGCTTGCGTGA

>TaSOD2.2

ATGGCGCTCCGCACGCTGGCCGCGAAGAAAACCCTAGGCCTGGCGCTCGGCGGCGCCAGGGGCGTGGCGACGTTCACGCTCCCCGACCTCCCCTACGACTACGGCGCGCTGGAGCCGGCCGTCTCCGGCGAGATCATGCGCCTGCACCACCAGAAGCACCACGCCACCTACGTCGCCAACTACAACAAGGCGCTCGAGCAGCTCGACGCCGCCGCCAGCAAGGGGGACGCGTCCGCTGTCGTCGGCCTCCAGAGCGCCATCAAGTTCAACGGCGGCGGTCATGTTAACCATTCAATCTTCTGGAAGAACCTCAAGCCCATTAGCGAGGGTGGTGGTGAGGCACCTCATGG

CAAACTTGGCTGGGCCATTGATGAGGATTTTGGTTCTATTGAGAAACTTATAAAGAAGATGAATGCAGAGGGTGCTGCTTTACAAGGATCTGGATGGGTGTGGCTAGCTTTGGATAAAGAGGCCAAAAGGCTTTCAGTTGAAACTACTCCTAATCAGGACCCTCTTGTGACCAAAGGGTCAAACCTGCATCCTTTGTTGGGAATTGATGTCTGGGAGCATGCGTACTACCTGCAGTACAAGAACGTGAGGCCGGACTACCTGACCAACATCTGGAAGGTGGTGAACTGGAAATACGCTGGAGAAGAGTATGAAAAAGTGCTTGCGTGA

>TaSOD2.3

ATGGCGCTCCGCACGTTGGCCGCGAAGAAAACCCTAGGCCTGGCGCTCGGCGGCGCGCGGCCGCCGGCCGCCGCCAGGGGCGTGGCGACGTTCACGCTCCCCGACCTCCCCTACGACTACGGCGCGCTGGAGCCGGCCGTCTCCGGCGAGATCATGCGCCTGCACCACCAGAAGCACCACGCCACCTACGTCGCCAACTACAACAAGGCGCTCGAGCAGCTCGACGCCGCCGTCAGCAAGGGGGACGCGTCCGCCGTCGTCCACCTCCAGAGCGCCATCAAGTTCAACGGCGGCGGTCATGTTAACCATTCAATCTTCTGGAAGAACCTCAAGCCCATCAGCGAGGGTGG

TGGTGAGGCACCTCATGGCAAACTTGGCTGGGCCATTGATGAGGATTTTGGTTCTATTGAGAAACTTATAAAGAAGATGAATGCAGAGGGTGCTGCTTTACAAGGATCTGGATGGGTGTGGCTAGCTTTGGATAAAGAGGCCAAAAGGCTTTCAGTTGAAACTACTCCTAATCAGGACCCTCTTGTGACCAAAGGGTCAAACCTGCATCCTTTGTTGGGAATTGATGTCTGGGAGCATGCGTACTACCTGCAGTACAAGAACGTGAGGCCGGACTACCTGACCAACATCTGGAAGGTGGTGAACTGGAAATATGCCGGAGAAGAGTATGAGAAAGTGCTTGCATGA

>TaSOD2.4

ATGCTCCTCCCAATGCGCGGCCTCCCGGCCGCCCCACCCCGCCCGCTCGCCCACCCACACGCCTCCCCCGCGCCGCCGCCGCCCTCCCTCCTCAGTCCCCGGCGCCGCCGGCCGTCCCGGAGGCTCTCCAAGGTCGTGTCCTACTACGGCCTCACGACCCCGCCGTACAAAACCGACGCCCTGGAGCCGTACATGAGCAGGCGGGCGGTGGAGCTGCACTGGGGCAAGCACCAGCAGGGCCACGTGGACGGGCTCAACAAGCAGCTGGCCATCAGCCCGCTCTACGGCCACACCCTCGAGGACCTCATCAAGGAGGCCTACAACAACGGCAACCCGCTGCCCGAGTACAACGACGCGGCCGAGGTCTGGAACCATCACTTCTTCTGGGAGTCGATGCAGCCGGACGGCGGCGGCTCGCCCGAGGCGGGCGTGCTGCAGCAGATCGAGAAGGACTTCGGCTCCTTTTTTAATTTCAGGGAGGAGTTCATGCGCTCGGCGCTGTCGTTGTTGGGGTCCGGTTGGGTTTGGCTTGTCTTGAAGAGGAGCGAGAGGAAGCTCGAGGTGGTTCACACGCGAAATGCTATCAACCCACTTGCTTTTGGAGACATTCCAATCATCAGCCTAGACTTGTGGGAGCATGCTTACTACTTAGATTACAAGGATGACAGGCGAACGTATGTGTCAAACTTTCTGGATCACCTTGTGTCTTGGCATACTGTCACTCTACGCATGATGCGCGCGGAGGCTTTTGTGAACCTTGGTGAACCAACTATCCCAGTGGCATGA

>TaSOD2.5

ATGGCGTTCGCCGCGCCGGTAGGGGTGGGAGGCGGCCCCCTCTCCCTCGCCCTCCCCGCCTCCAGCTCGGCCCCCTTCCTCCTCCGCGCCGGCGGCGACTCCCCGCAGCGTGGGAGGCTCCGCCGCCTCGCCGCCCCACGGAGAGGAGGCGCCAGGGGAGATAGCCGGGGGAGATGGAATTGCCACGTAACTCGCTGTGCCGGCGAGGCGAATGTGGTGACCGAGGACGATACGGCGAATGTTGCTGCTGATGCCGCAGCGGACCAAGCTGCCAATGCCAGCGGCGATGCCGCCGATGTGTCCTTGAACCCTGACGACGTTGATTCAGTTGCCTGGATAAAGCAGCAGCC

TCTCCCTTATCCTGCAGATGCTCTGGAGCCGTACATAAGCAAGGAGACGGTGGAGCAGCACTGGGGAGTTCACCAGCGCGGGCACGTGGACAGGCTCAACGGCATGATCGGCGGCAGCGAGTGGGAGCGGATGTCGATTGGGCAGATGATGCTCGCCTCATTCAATGAGGGCAGGGAGCCACCCCATGCTCCCTTCTTCCATGCCGCACAGGTATGGAACCATGATTTCTATTGGCGCTCGATAAAACCTGGCGGTGGAGGCAAGCCCCCGGAACGTCTTCTGAAGTTTATAAACAGGGACTTCGGATCCTATGACGGCATGATCAAGCAATTCATGGATGTCGCACTAACTCAGTTCGGTTCTGGATGGGTCTGGCTTTCTTACAAGGGGAGCAAGTTACCCCATGTGAAGTCAAAAAGCCCAATCCCATCTGACAATTATGGTAGGCTGGTCATCTCAAAGTCTCCAAATGCTATCAACCCTCTTGTCTGGGGCCACTCTCCACTCCTTGCGATTGATGTTTGGGAGCATGCCTACTACTTGGATTATGAGAATCGAA

GGGCTGAGTATGTCTCTGCAGTTCTAGAGAAGCTCGTCTCATGGGAAATGGTGGAGTCCAGGCTCCGGAAAGCTGTTCTGCGAGCAATAGAAAGAGACGGTCACACCAGCCCGAAGCAGCGAAGGAAGCAACTTCTGTCCCAGGCTAAAAGCCGCGTCGGGGATGCAAGCACCAGCGGTGAGGCAAGAAGGCGACCAAGAAGTAAGGATCAGCAGGCCCCTTCCAGCGTGAGAATGGTTCCAGCAGGCGAGGCGGTACCGAACTGA

>TaSOD2.6

ATGGTGTTCGCCGCGCCGGCAGGGGTGGGAGGCCGCCCCCTCTCCCTCGCTCTCCCCGCCTCCAGCTCGGCCCCCTTCCTCCTCCGCGACGGCGGCGACTCCCCGCAGCGTGGGAGGCTGCGCCGCCTCACCTTCCCACGGAGAGGAGGCGCCAGGGGAGATAGCCGGGGGAGATGGAATTGCCACATAACTCGCTGTGCCGGCGAGGCGAATGTGGTGACCGAGGATGATACGGCGAATGTTTCTGCTGATGCCGCTGCGGACAAAGCCGCCGATGCCATCGGCGATGGCGCGGATGTGCTGGAGTCCTTGAACCCTGACAATGCTGATTCAGTTGCCTGGATAAAGCAGCAGCCTCTCCCTTATCCTTCAGATGCTCTGGAGCCATACATAAGCAAGGAGACGGTGGCGCAGCACTGGGGAGTTCACCAGCACATGCACGTGGACAGGCTCAACGGCATGATCGGCGGCAGCGAGTGGGAGCGCATGTCGATTGGGCAGATGATGCTTGCCTCGTTCAACGAGGGCAGGGAGCCACCCCACGCTCCCTTCTTCCATGCCGCACAGGTATGGAACCATGATTTCTACTGGCGATCGATGAAGCCTGGCGGTGGAGGCAAGCCCCCCGAACGGCTTCTGAAGTTTATAAACAGAGACTTCGGATCCTATGACGGCATGATCAAACAATTCATGGATGCCGCGCTAACTCAGTTCGGTTCTGGATGGGTTTGGCTTTCTTACAAGGGGAGCAAGTTACCTCATGTGAAGTCAAAAAGCCCAATCCCATCTGACAACTATGGTAGGCTGGTCATCTCAAAGTCTCCAAATGCTATCAATCCTCTTGTCTGGGGCCACTCTCCACTCCTTGCGATTGATGTTTGGGAGCATGCCTACTACCTGGATTATGAGAATCGAAGGGCTGAGTACGTCTCTGCAGTTCTAGAGAAGCTCGTGTCATGGGAAATGGTGGAGTCCAGGCTCCGGAAAGCTGTTCTGCGAGCAATAGAGAGAGACGGCCACACCAGCCCGAAGCAGCGAAGGAAGCAACTTCTGTCTCAGGCTAGAAGCCGCGTTGGGGATGCAAGCACCAGCGGTGAGGCAAGAAGGCGACCAAGAAGTAAGGATCAGCAGGCCCCTTCCAGCGTGACAATGGTGCCAGCAGGCGAGGCAGTACCGAACTGA

>TaSOD2.7

ATGCTCCTCCCAATGCGCGGCCTCCCGGCCGCCCCACCCCGCCCGCTCGCCCACCCACACACCGCGCCCGCGCCGCCGCCCTCGCTCCTCGGTCCCCGGCGCCGCCGTCCGTCCCGGAGGCTCTCCAAGGTCGTGTCCTACTACGGCCTCACGACTCCGCCGTACAAAACCGACGCCCTGGAGCCGTACATGAGCAGGCGGGCGGTGGAGCTGCACTGGGGCAAGCACCAGCAGGACTACGTGGACGGGCTCAACAAGCAGCTCGCCATCAGCCCGCTCTACGGCCACACCCTCGAGGACCTCATCAAGGAGGCCTACAACAACGGCAACCCGCTGCCCGAGTACAACGACGCGGCCGAGGTCTGGAACCATCACTTCTTCTGGGAATCGATGCAGCCGGAAGGCGGCGGCTCGCCCGAGGCAGGTGTGCTGCAGCAGATCGAGAAGGATTTCGGCTCCTTTTTTAATTTCAGGGAGGAGTTCATGCGCTCGGCGTTGTCGTTGTTGGGGTCTGGTTGGGTTTGGCTTGTCTTGAAGAGAAGCGAGAGGAAGCTTGAGGTGGTTCACACGCGAAACGCTATCAACCCACTTGCTTTTGGAGATATTCCAATCATCAGCCTAGACTTGTGGGAGCATGCTTACTACTTGGATTACAAGGATGACAGGCGAACATATGTGTCAAACTTTCTGGATCACCTTGTCTCTTGGCATACTGTCACTCTACGCATGATGCGCGCGGAGGCTTTTGTGAACCTTGGTGAACCAACTATCCCAGTGGCATGA

>TaSOD2.8

ATGGTCTTCGCCGCGCCGGCAGGGGTGGGAGGCGGCCCCCTCTCCGTCGCTCCCCCCGCCTCCAGCTCGGCCCCCTTCCTCCTCCGCGCCGGCGGCGACTCGCCGCAGCGCGGGATGCTCCGCCGCCTCACCTTCCCACGGAGAGGAGGCGCCAGGGGACAGAGCCAGAGAAGATGGGATTGCCACATAACTCGCTGTGCCGGCAAGGCGAATGTGGTGACCGAGGATGATACGGCGAAAGTTGCTGCTGCTGATGCCACCGCAGACCAAGCTGCCGATGATACCGCCGATGTGCTCGAGTCCTTGAACCCTGACGACGACGTTGATTCAGTTGCCTGGATAAAGCAGCAGCCCCTCCCTTATCCTGCAGATGCTCTGGAGCCGTACATAAGCAAGGAGACGGTGGAGCAGCACTGGGGAGTTCACCAGCGCATGCATGTGGACAGGCTCAACGGCATGATCGGCGGCAGCGAGTGGGAGCGGATGTCGATTGGGCAGATGATGCTCGCCTCATTCAACGAGGGCAGGGAGCCACCCCACGCTCCCTTCTTCCATGCCGCACAGGTATGGAACCATGATTTCTACTGGCGATCGATGAAACCTGGCGGTGGAGGCAAGCCCCCGGAACGTCTTCTGAAGTTTATTAACAGGGACTTCGGATCCTATGACGGCATGATCAAACAATTCATGGATGCGGCATTAACTCAGTTCGGTTCTGGATGGGTTTGGCTTTCTTACAAGGGGAGCAAGTTACCTCATGTGAAGTCAAAAAGCCCAATCCCATCTGACAATTATGGTAGGCTGGTCATCTCAAAGTCTCCGAATGCTATCAACCCTCTTGTCTGGGGCCACTCTCCACTCCTTGCGATTGATGTTTGGGAGCATGCCTACTACCTGGATTATGAGAATCGAAGGGCTGAGTATGTCTCTGCAGTTCTAGAGAAGCTCGTGTCATGGGAAATGGTGGAGTCCAGGCTCCGGAAAGCTGTTCTGCGAGCAATAGAAAGAGACGGTCACACCAGCCCGAAGCAGCGAAGGAAGCAACTTGTGTCCCAGGCTAAAGGCCGCGTCGGGGATGCAAGCACCAGCGGTGAGGCAAGACGGCGACCGAGAAGTAAGGATCAGCAGGCCCCTTCCAGCGTGACAATGGTGCCAGCAGGCGAGGCGGTACCGAACTGA

>TaSOD2.9

ATGCTCCTCCCAGTGCGCGGCCTCCCGGCCGCCCCTCATCGCCCGCTCACCCACCCACACAGCTCCCCCGCGCCGCCGCCCTCCCTCCTCGGTGCCCGGCGCCGCCGTCCGTCCCGGAGGCTCTCCAAGGTCGTGTCCTACTACGGCCTCACCACTCCGCCGTACAAAACCGACGCCCTGGAGCCGTACATGAGCAGGCGGGCGGTGGAGCTGCACTGGGGCAAGCACCAGCAGGAGTACGTGGACGGGCTCAACAGGCAGCTCGCCATCAGCCCGCTCTACGGCCACACCCTCGAGGACCTCATCAAGGAGGCCTACAACAACGGCAACCCGCTGCCCGAGTACAACGACGCGGCCGAGGTCTGGAACCATCACTTCTTCTGGGAATCGATGCAGCCGGAAGGCGGCGGCTCGCCCGAGGCGGGCGTGCTGCAGCAGATCGAGAAGGATTTCGGCTCCTTTTTTAATTTCAGGGAGGAGTTCATGCGCTCGGCGTTGTCGTTGTTGGGGTCTGGTTGGGTTTGGCTTGTCTTGAAGAGAAGCGAGAGGAAGCTCGAGGTGGTTCACACCCGAAATGCTATCAACCCACTTGCTTTTGGGGATATTCCAATCATCAGCCTAGACTTGTGGGAGCATGCTTACTACTTAGATTACAAGGATGACAGGCGAACATATGTGTCAAACTTTCTGGATCACCTTGTGTCTTGGCATACTGTCACTGTACGCATGATGCGCGCGGAGGCTTTTGTTAACCTTGGTGAACCAACTATCCCAGTGGCATGA

1. **Protein sequences of wheat SOD**

>TaSOD1.1a

QITHTMVKAVAVLTGSEGVKGTIFFTQEGEGPTTVTGSVTGLKEGLHGFHVHALGDTTNGCMSTGPHFNPAGHVHGAPEDEIRHAGDLGNVTAGVDGVASINITDCHIPLTGPNSIVGRAVVVHGDADDLGKGGHELSKSTGNAGARVACGIIGLQG*

>TaSOD1.1b

MVKAVAVLTGSEGVKGTIFFTQEGEGPTTVTGSVTGLKEGLHGFHVHALGDTTNGCMSTGPHFNPAGHVHGAPEDEIRHAGVASINITDCHIPLTGPNSIVGRAVVVHGDADDLGKGGHELSKSTGNAGARVACGIIGLQG*

>TaSOD1.2

MVGFLRAFTAASAVPAAAVAAAALSSSSSSPSRSPSSRLRFPLPPSLSAFAASSSSSAPV

RAPTAAPPMAAAATADLSAPDKGTALPELTTEFMVDMKCEGCVTAVKNRLQTLEGIQNIE

VDLNNQVVRVRGSLPVKIMLDALHQTGRDARLIGQGNPDDFLVSAAVAEFKGPVIFGVVR

LAQVNMELARVEATFSGLSPGKHGWSINEFGDLTKGAESTGKVYNPQDYLSDKPLGDLGT

LEAGENGEAQFSGSKEKLKVVDLIGRSIALYATEDRSDPGIAAAVVARSAGVGENYKKLC

TCDGVTIWESS*

>TaSOD1.3

MVGFLRAFTAASAVPAAAVAAVALSSSSPSRSPSSRLRFPLPPSLSAFAASSASSSPVRA

PTAAPPMAAAATADLSAPDKGTALPELTTEFMVDMKCEGCVTAVKNKLQTLEGIQNIEVD

FNNQVVRVRGSLPVKLMLDALRQTGRDARLIGQGNPDDFLVSAAVAEFKGPVIFGVVRLA

QVNMELARVEATFSGLSPGKHGWSINEFGDLTKGAESTGKVYNPQDYLSDKLLGDLGTLE

AGEREAQFSGSKEKLKVVDLIGRSIALYATEDRSDAGLAAAVVARSAGVGENYKKLCTCD

GVTIWESS*

>TaSOD1.4

MVKAVAVLTGSEGVKGTIFFTQEGDGPTTVTGSVTGLKEGLHGFHVHALGDTTNGCMSTG

PHFNPAGHVHGAPEDEIRHAGDLGNVTAGADGVANINVTDCHIPLTGPNSIVGRAVVVHG

DADDLGKGGHELSKSTGNAGARVACGIIGLQG*

>TaSOD1.5a

MVGFLRAFTAASAVPAAAVAAAALCSSSSPSRSPSSRLRFPLPSLSAFAASSSSSSPVRA

PTAAPPMAAAATADLSAPDKGTALPELTTEFMVDMKCEGCVTAVKNRLQTLEGIQNIEVD

LNNQVVRVRGSLPVKIMLDALHQTGRDARLIGQGNPDDFLVSAAVAEFKGPVIFGVVRLA

QVNMELARIEATFSGLSPGKHGWSINEFGDLTKGAESTGKVYNPQDYLSDKPLGDLGTLE

AGENGEAQFSGSKEKLKVVDLIGRSIALYATEDRSDPGIAAAVVARSAGVGENYKKLCTC

DGVTIWESS*

>TaSOD1.5b

MVGFLRAFTAASAVPAAAVAAAALCSSSSPSRSPSSRLRFPLPSLSAFAASSSSSSPVRA

PTAAPPMAAAATADLSAPDKTEFMVDMKCEGCVTAVKNRLQTLEGIQNIEVDLNNQVVRV

RGSLPVKIMLDALHQTGRDARLIGQGNPDDFLVSAAVAEFKGPVIFGVVRLAQVNMELAR

IEATFSGLSPGKHGWSINEFGDLTKGAESTGKVYNPQDYLSDKPLGDLGTLEAGENGEAQ

FSGSKEKLKVVDLIGRSIALYATEDRSDPGIAAAVVARSAGVGENYKKLCTCDGVTIWES

S*

>TaSOD1.6a

MAGKPGSLKGVALISGGGADSAVAGALHFVQDPSSGYTEVRGRVSGLAPGLHGFHIHAFG

DTTNGCNSTGPHFNPHNKSHGAPVDDERHVGDLGNIQANKDGVAEIFIKDLQISLRGPHS

ILGRAVVVHADSDDLGKGGHELSKSTGNAGARIGCGIIGIQPAV*

>TaSOD1.6b

MAGKPGSLKGVALISGGGADSAVAGALHFVQDPSSGYTEVRGRVSGLAPGLHGFHIHAFG

DTTNGCNSTGPHFNPHNKSHGAPVDDERHVGDLGNIQANKDGVAEIFIKDLQISLRGPHS

ILGRAVVVHADSDDLGKGGHELSKSTGNAGARIGCGKWQTLYLFSTLSKCTHKVSLEFSR

LFNKKNTRAVGVHVGIAARRQGNSDIVEDHTF*

>TaSOD1.7a

MAGKPGSLKGVALISGGSADSAVAGALHFVEDPSSGYTEVRGRVSGLAPGLHGFHIHAFGDTTNGCNSTGPHFNPHNKFHGAPMDDERHVGDLGNIQANKDGVAEIFIKDLQISLRGPHSILGRAVVVHADSDDLGKGGHELSKSTGNAGARIGCGVIGIQPAV*

>TaSOD1.7b

MAGKPGSLKGVALISGGSADSAVAGALHFVEDPSSGYTEVRGRVSGLAPGLHGFHIHAFGDTTNGCNSTGPHFNPHNKFHGAPMDDERHVGDLGNIQANKDGVAEIFIKDLQISLRGPHSILGRAVVVHADSDDLGKGGHELSKSTGNAGARIGCGKWQTLHLFSTLSKCTHKV*

>TaSOD1.8a

MAGKPVSLKGVALISGGAADSAVAGALHFVQDPSSGYTEVRGWVSGLAPGLHGFHIHAFGDTTNGCNSTGPHFNPHNKSHGAPVDDERHVGDLGNIQANKDGVAEIFIKDLQISLRGPHSILGRAVVVHADSDDLGKGPWVTLDHD*

>TaSOD1.8b

MAGKPVSLKGVALISGGAADSAVAGALHFVQDPSSGYTEVRGWVSGLAPGLHGFHIHAFGDTTNGCNSTGPHFNPHNKSHGAPVDDERHVGDLGNIQANKDGVAEIFIKDLQISLRGPHSILGRAVVVHADSDDLGKGGHELSKSTGNAGARIGCGIIGIQPAV*

>TaSOD1.9

MAAQSLLFAAAAPLFQAPASARPFQSLRIVCTPEGATAAARALVVADATKKAVAVLKGTSQVEGVVTLTQEDDGPTTVNVRITGLAPGLHGFHLHEFGDTTNGCISTGPHFNPNGLTHGAPEDEVRHAGDLGNIVANAEGVAETTIVDSQIPLTGPNAVVGRAFVVHELEDDLGKGGHELSLSTGNAGGRLACGVVGLTPL*

>TaSOD1.10

MAAQSLLFAAAAPLFQVPASARPFQSLRIVSTPGGATAAARALVVADATKKAVAVLKGTSQVEGVVTLTQEDDGPTTVNVRITGLAPGLHGFHLHEFGDMTNGCISTGPHFNPNGLTHGAPEDEVRHAGDLGNIVANAEGVAETTIVDSQIPLTGPNAVVGRAFVVHELEDDLGKGGHELSLSTGNAGGRLACGVVGLTPL*

>TaSOD1.11a

MAAQSLLFAAAAPLFQAPASARPFQSLRIVSTPGGATAAARALVVADATKKAVAVLKGSSQVEGVVTLTQEDDGPTTVNVRITGLAPGLHGFHLHEFGDTTNGCISTGPHFNPNGLTHGAPEDEVRHAGDLGNIVANAEGVAETTIVDSQIPLTGPNAVVGRAFVVHELEDDLGKGGHELSLSTGNAGGRLACGVVGLTPL*

>TaSOD1.11b

MAAQSLLFAAAAPLFQAPASARPFQSLRIVSTPGGATAAARALVVADATKKAVAVLKGSSQVEGVVTLTQEDDAGPTTVNVRITGLAPGLHGFHLHEFGDTTNGCISTGPHFNPNGLTHGAPEDEVRHAGDLGNIVANAEGVAETTIVDSQIPLTGPNAVVGRAFVVHELEDDLGKGGHELSLSTGNAGGRLACGVVGLTPL*

>TaSOD2.1

MALRTLAAKKTLGLALGGARPLAAARGVATFTLPDLPYDFGALEPAVSGEIMRLHHQKHHATYVANYNKALEQLDAAVSKGDASAVVHLQSAIKFNGGGHVNHSIFWKNLKPISEGGGEPPHGKLGWAIDEDFGSIEKLIKKMNAEGAALQGSGWVWLALDKEAKKLSVETTPNQDPLVTKGSNLYPLLGIDVWEHAYYLQYKNVRPDYLTNIWKVVNWKYAGEEYEKVLA*

>TaSOD2.2

MALRTLAAKKTLGLALGGARGVATFTLPDLPYDYGALEPAVSGEIMRLHHQKHHATYVANYNKALEQLDAAASKGDASAVVGLQSAIKFNGGGHVNHSIFWKNLKPISEGGGEAPHGKLGWAIDEDFGSIEKLIKKMNAEGAALQGSGWVWLALDKEAKRLSVETTPNQDPLVTKGSNLHPLLGIDVWEHAYYLQYKNVRPDYLTNIWKVVNWKYAGEEYEKVLA*

>TaSOD2.3

MALRTLAAKKTLGLALGGARPPAAARGVATFTLPDLPYDYGALEPAVSGEIMRLHHQKHHATYVANYNKALEQLDAAVSKGDASAVVHLQSAIKFNGGGHVNHSIFWKNLKPISEGGGEAPHGKLGWAIDEDFGSIEKLIKKMNAEGAALQGSGWVWLALDKEAKRLSVETTPNQDPLVTKGSNLHPLLGIDVWEHAYYLQYKNVRPDYLTNIWKVVNWKYAGEEYEKVLA*

>TaSOD2.4

MLLPMRGLPAAPPRPLAHPHASPAPPPPSLLSPRRRRPSRRLSKVVSYYGLTTPPYKTDALEPYMSRRAVELHWGKHQQGHVDGLNKQLAISPLYGHTLEDLIKEAYNNGNPLPEYNDAAEVWNHHFFWESMQPDGGGSPEAGVLQQIEKDFGSFFNFREEFMRSALSLLGSGWVWLVLKRSERKLEVVHTRNAINPLAFGDIPIISLDLWEHAYYLDYKDDRRTYVSNFLDHLVSWHTVTLRMMRAEAFVNLGEPTIPVA*

>TaSOD2.5

MAFAAPVGVGGGPLSLALPASSSAPFLLRAGGDSPQRGRLRRLAAPRRGGARGDSRGRWNCHVTRCAGEANVVTEDDTANVAADAAADQAANASGDAADVSLNPDDVDSVAWIKQQPLPYPADALEPYISKETVEQHWGVHQRGHVDRLNGMIGGSEWERMSIGQMMLASFNEGREPPHAPFFHAAQVWNHDFYWRSIKPGGGGKPPERLLKFINRDFGSYDGMIKQFMDVALTQFGSGWVWLSYKGSKLPHVKSKSPIPSDNYGRLVISKSPNAINPLVWGHSPLLAIDVWEHAYYLDYENRRAEYVSAVLEKLVSWEMVESRLRKAVLRAIERDGHTSPKQRRKQLLSQAKSRVGDASTSGEARRRPRSKDQQAPSSVRMVPAGEAVPN*

>TaSOD2.6

MVFAAPAGVGGRPLSLALPASSSAPFLLRDGGDSPQRGRLRRLTFPRRGGARGDSRGRWNCHITRCAGEANVVTEDDTANVSADAAADKAADAIGDGADVLESLNPDNADSVAWIKQQPLPYPSDALEPYISKETVAQHWGVHQHMHVDRLNGMIGGSEWERMSIGQMMLASFNEGREPPHAPFFHAAQVWNHDFYWRSMKPGGGGKPPERLLKFINRDFGSYDGMIKQFMDAALTQFGSGWVWLSYKGSKLPHVKSKSPIPSDNYGRLVISKSPNAINPLVWGHSPLLAIDVWEHAYYLDYENRRAEYVSAVLEKLVSWEMVESRLRKAVLRAIERDGHTSPKQRRKQLLSQARSRVGDASTSGEARRRPRSKDQQAPSSVTMVPAGEAVPN*

>TaSOD2.7

MLLPMRGLPAAPPRPLAHPHTAPAPPPSLLGPRRRRPSRRLSKVVSYYGLTTPPYKTDALEPYMSRRAVELHWGKHQQDYVDGLNKQLAISPLYGHTLEDLIKEAYNNGNPLPEYNDAAEVWNHHFFWESMQPEGGGSPEAGVLQQIEKDFGSFFNFREEFMRSALSLLGSGWVWLVLKRSERKLEVVHTRNAINPLAFGDIPIISLDLWEHAYYLDYKDDRRTYVSNFLDHLVSWHTVTLRMMRAEAFVNLGEPTIPVA*

>TaSOD2.8

MVFAAPAGVGGGPLSVAPPASSSAPFLLRAGGDSPQRGMLRRLTFPRRGGARGQSQRRWDCHITRCAGKANVVTEDDTAKVAAADATADQAADDTADVLESLNPDDDVDSVAWIKQQPLPYPADALEPYISKETVEQHWGVHQRMHVDRLNGMIGGSEWERMSIGQMMLASFNEGREPPHAPFFHAAQVWNHDFYWRSMKPGGGGKPPERLLKFINRDFGSYDGMIKQFMDAALTQFGSGWVWLSYKGSKLPHVKSKSPIPSDNYGRLVISKSPNAINPLVWGHSPLLAIDVWEHAYYLDYENRRAEYVSAVLEKLVSWEMVESRLRKAVLRAIERDGHTSPKQRRKQLVSQAKGRVGDASTSGEARRRPRSKDQQAPSSVTMVPAGEAVPN*

>TaSOD2.9

MLLPVRGLPAAPHRPLTHPHSSPAPPPSLLGARRRRPSRRLSKVVSYYGLTTPPYKTDALEPYMSRRAVELHWGKHQQEYVDGLNRQLAISPLYGHTLEDLIKEAYNNGNPLPEYNDAAEVWNHHFFWESMQPEGGGSPEAGVLQQIEKDFGSFFNFREEFMRSALSLLGSGWVWLVLKRSERKLEVVHTRNAINPLAFGDIPIISLDLWEHAYYLDYKDDRRTYVSNFLDHLVSWHTVTVRMMRAEAFVNLGEPTIPVA*

1. **Protein sequence of Arabidopsis, rice, and maize.**

>AT1G08830.1

MAKGVAVLNSSEGVTGTIFFTQEGDGVTTVSGTVSGLKPGLHGFHVHALGDTTNGCMSTGPHFNPDGKTHGAPEDANRHAGDLGNITVGDDGTATFTITDCQIPLTGPNSIVGRAVVVHADPDDLGKGGHELSLATGNAGGRVACGIIGLQG

>AT2G28190.1

MAATNTILAFSSPSRLLIPPSSNPSTLRSSFRGVSLNNNNLHRLQSVSFAVKAPSKALTVVSAAKKAVAVLKGTSDVEGVVTLTQDDSGPTTVNVRITGLTPGPHGFHLHEFGDTTNGCISTGPHFNPNNMTHGAPEDECRHAGDLGNINANADGVAETTIVDNQIPLTGPNSVVGRAFVVHELKDDLGKGGHELSLTTGNAGGRLACGVIGLTPL

>AT3G10920.1

MAIRCVASRKTLAGLKETSSRLLRIRGIQTFTLPDLPYDYGALEPAISGEIMQIHHQKHHQAYVTNYNNALEQLDQAVNKGDASTVVKLQSAIKFNGGGHVNHSIFWKNLAPSSEGGGEPPKGSLGSAIDAHFGSLEGLVKKMSAEGAAVQGSGWVWLGLDKELKKLVVDTTANQDPLVTKGGSLVPLVGIDVWEHAYYLQYKNVRPEYLKNVWKVINWKYASEVYEKENN

>AT3G56350.1

MTTTVIIIIFVAIFATTLHDARGATMEPCLESMKTASLPDLPYAYDALEPAISEEIMRLHHQKHHQTYVTQYNKALNSLRSAMADGDHSSVVKLQSLIKFNGGGHVNHAIFWKNLAPVHEGGGKPPHDPLASAIDAHFGSLEGLIQKMNAEGAAVQGSGWVWFGLDRELKRLVVETTANQDPLVTKGSHLVPLIGIDVWEHAYYPQYKNARAEYLKNIWTVINWKYAADVFEKHTRDLDIN

>AT4G25100.1

MAASSAVTANYVLKPPPFALDALEPHMSKQTLEFHWGKHHRAYVDNLKKQVLGTELEGKPLEHIIHSTYNNGDLLPAFNNAAQAWNHEFFWESMKPGGGGKPSGELLALLERDFTSYEKFYEEFNAAAATQFGAGWAWLAYSNEKLKVVKTPNAVNPLVLGSFPLLTIDVWEHAYYLDFQNRRPDYIKTFMTNLVSWEAVSARLEAAKAASA

>AT5G18100.1

MEAPRGNLRAVALIAGDNNVRGCLQFVQDISGTTHVTGKISGLSPGFHGFHIHSFGDTTNGCISTGPHFNPLNRVHGPPNEEERHAGDLGNILAGSNGVAEILIKDKHIPLSGQYSILGRAVVVHADPDDLGKGGHKLSKSTGNAGSRVGCGIIGLQSSADAKL

>AT5G23310.1

MSSCVVTTSCFYTISDSSIRLKSPKLLNLSNQQRRRSLRSRGGLKVEAYYGLKTPPYPLDALEPYMSRRTLEVHWGKHHRGYVDNLNKQLGKDDRLYGYTMEELIKATYNNGNPLPEFNNAAQVYNHDFFWESMQPGGGDTPQKGVLEQIDKDFGSFTNFREKFTNAALTQFGSGWVWLVLKREERRLEVVKTSNAINPLVWDDIPIICVDVWEHSYYLDYKNDRAKYINTFLNHLVSWNAAMSRMARAEAFVNLGEPNIPIA

>AT5G51100.1

MMNVAVTATPSSLLYSPLLLPSQGPNRRMQWKRNGKRRLGTKVAVSGVITAGFELKPPPYPLDALEPHMSRETLDYHWGKHHKTYVENLNKQILGTDLDALSLEEVVLLSYNKGNMLPAFNNAAQAWNHEFFWESIQPGGGGKPTGELLRLIERDFGSFEEFLERFKSAAASNFGSGWTWLAYKANRLDVANAVNPLPKEEDKKLVIVKTPNAVNPLVWDYSPLLTIDTWEHAYYLDFENRRAEYINTFMEKLVSWETVSTRLESAIARAVQREQEGTETEDEENPDDEVPEVYLDSDIDVSEVD

>LOC_Os03g11960.1

MAGKAGGLKGVALIGGAGGNSAVAGALHFFQDPSTGYTEVRGRVTGLAPGLHGFHIHSFGDTTNGCNSTGPHFNPHNKSHGAPSDDERHVGDLGNIVANKDGVADIFIKDLQISLSGPHSILGRAVVVHADSDDLGRGGHELSKTTGNAGARIGCGIIGLRSAV*

>LOC_Os03g22810.1

MVQVISDELRLSPLTGRALNTGQRRIYPVVPNYLDTGSSAKELGVTERRQSVTLGARALNVGWWLQLQAASLTTNTTAHRWREGNLPEAPDSKPAGVASPPPSSSSSSPRGSPEITLTMVKAVVVLGSSEIVKGTIHFVQEGDGPTTVTGSVSGLKPGLHGFHIHALGDTTNGCMSTGPHYNPAGKEHGAPEDETRHAGDLGNVTAGEDGVANIHVVDSQIPLTGPNSIIGRAVVVHADPDDLGKGGHELSKTTGNAGGRVACGIIGLQG*

>LOC_Os04g48410.1

MVGFLRALTAASAVPAAAAVAAVALSTNSSSSSRLRLPSPASLPSLSSAYAAAPASGSARKPNAVPPMAAAAATADLSAAADKGAALPELMTEFMVDMKCDGCVTAVKNKFQTLEGIKNIEVDLNNQVVRVLGSLPVNTMLDTLHQTGRDARLIGQGNPNDFLVSAAVAEFKGPVIFGVVRLAQVNMELAIVEATFSGLSPGKHGWSINEFGDLTRGAESTGKVYNPSDYRSNKPLGDLGTLEAGEKGEAQFSASKEKLKVVDLIGRSIALYATEDRSDPGIAAAVIARSAGVGENYKKLCTCDGVTIWESS*

>LOC_Os05g25850.1

MALRTLASRKTLAAAALPLAAAAAARGVTTVALPDLPYDYGALEPAISGEIMRLHHQKHHATYVANYNKALEQLDAAVAKGDAPAIVHLQSAIKFNGGGHVNHSIFWNNLKPISEGGGDPPHAKLGWAIDEDFGSFEALVKKMSAEGAALQGSGWVWLALDKEAKKLSVETTANQDPLVTKGANLVPLLGIDVWEHAYYLQYKNVRPDYLSNIWKVMNWKYAGEVYENATA*

>LOC_Os06g02500.1

MAFATLVGVGGLSPALFSPSRPLSCSSSTSVSAPFILRAGGGGDARRHGLRRLVTPLRGSACRGESTNSRVLQCANEANVVTEDDIVNDGIDDETASDAEMDEDAEANGDESSGTDEDASVSWIEQQPLPYPSDALEPYISKETVEQHWGVHQNIHVERLNGMIGGSEWEGMSLGQMMLSSFNEGREAPHPPFFHAAQIWNHDFYWRSMQPGGGGKPPERLLKFINRDFGSYDGMIRQFMDAASTQFGSGWVWLCYKTSKLPHVKSRSPIPSDNYGRLVISKSPNAINPLVWGHSPLLAIDLWEHAYYLDYEDRRSDYVSTFLEKLVSWETVESRLKKAVQRAVERDEYVSTKHIRKQLLARAKSQIRAMPQQVNGDAREQTSGQEKSLGV*

>LOC_Os06g05110.1

MAAFASALRVLPSPPAAVPRRLRSREQRQGCRSRRYSKVVAYYALTTPPYKLDALEPYISKRTVELHWGKHQQDYVDSLNKQLATSMFYGYTLEELIKEAYNNGNPLPEYNNAAQVWNHHFFWESMQPEGGGSPGRGVLQQIEKDFGSFTNFREEFIRSALSLLGSGWVWLVLKRKERKFSVVHTQNAISPLALGDIPLINLDLWEHAYYLDYKDDRRMYVTNFIDHLVSWDTVTLRMMRAEAFVNLGEPNIPVA*

>LOC_Os07g46990.1

MVKAVAVLASSEGVKGTIFFSQEGDGPTSVTGSVSGLKPGLHGFHVHALGDTTNGCMSTGPHFNPTGKEHGAPQDENRHAGDLGNITAGADGVANVNVSDSQIPLTGAHSIIGRAVVVHADPDDLGKGGHELSKTTGNAGGRVACGIIGLQG*

>LOC_Os08g44770.1

MQAILAAAMAAQTLLFSATAPPASLFQSPSSARPFHSLRLAAGPAGAAAARALVVADATKKAVAVLKGTSQVEGVVTLTQDDQGPTTVNVRVTGLTPGLHGFHLHEFGDTTNGCISTGPHFNPNNLTHGAPEDEVRHAGDLGNIVANAEGVAEATIVDKQIPLSGPNSVVGRAFVVHELEDDLGKGGHELSLSTGNAGGRLACGVVGLTPL*

>Zm00001d028232

MAGKAGGLKGVALIGGSANSTVAGVIHFFEDPSTSSRSLELSLSVFPMFIRIPTLPLARSPVAARVHRGEGQGHGLDSGKAWIPHPRLRRHYQWLQLNRFAAFFVYHGPHFNPHNKPHGAPFDDERHLGDLGNIVANEDGDAEVFIRDLQISLSGPHSILGRAVVVHADPDDLGRGKHACITIAIIL

>Zm00001d037859

MALRTLASKKVLSFPFGGAGRPLAAAASARGVTTVTLPDLSYDFIALEPAISGEIMRLHHQKHHATYVANYNKALEQLDTAVSKGDASAVVQLQGAIKFNGGGHVNHSIFWKNLKPISEGGGEPPHGKLGWAIDEDFGSFEALVKKMNAEGAALQGSGWVWLALDKEAKKLSVETTANQDPLVTKGASLVPLLGIDVWEHAYYLQVIFVMLLN

>Zm00001d029170

MSTGPHYNPASKEHGAPEDENRHAGDLGNVTAGADGVANINVTDSQIPLTGPNSIIGRAVVVHADPDDLGKDSLIITIYRSVILSDYSMAEREKIWWTRAQEEHRKRWRPCCLWYDHPFPSPILLRQTSFLFFFHTPRGFFIALYLPPQGSSDSRAELKQMVVGLRGRLGVPSPAG

>Zm00001d002611

MVDMKCEGCVTAVKNKLQTLEGIKNIEVDLSNQVVRVLGSLPVKTMLDALHQTGRDARLIGQGNPNDFLVSAAVAEFKGPVVFGVVRLAQVNMELARVEATFSGLSPGKHGWSINQFGDLTRGAESTGNVYNPPDHLSDKPLGDLGTLEAGENGEAHFSGPKEKLRVVDLIGRSIALYATEDRSDPGIAAAVIARSAGVGENYKKLCTCDGVTIWESS

>Zm00001d047479

MVKAVAVLGSSDGVKGTIFFTQEGDGPTAVTGSVSGLKPGLHGFHVHALGDTTNGCMSTGPHYNPASKEHGAPEDENRHAGDLGNVTAGADGVANINVTDSQIPLTGPNSIIGRAVVVHADPDDLGKGPIQSRSSKWSISDINSCWFSVCTFRWARAQQEHWKRRWPCCLWDHWTPGLKTIAGLGGA

>Zm00001d022505

MRSFVYAGYEIDQERRQIYAFFGPGNETVWDFVGKITETMVKAVAVLAGTDVKGTIFFSQEGDGPTTVTGSISGLKPGLHGFHVHALGDTTNGCMSTGPHFNPVGKEHGAPEDEDRHAGDLGNVTAGEDGVVNVNITDSQIPLAGPHSIIGRAVVVHADPDDLGKGGHELSKSTGNAGGRVACGKLYRGVFPVSWYHWAPRLKQLTSEANTIEGRSEHLRMLLFHFPCIGNTI

>Zm00001d036135

MAASALHLRLHPSPPAGVKLQQRKLRSKIQRQGGLSRRFSKVVSYYGLTTPPYKLDALEPYMSKRTVELHWGKHHQDYVDGLNKQLATSPLYGYTLEDLIKEAYNNGNPLPEYNNAAQVWNHHFFWESMQPEGGGLPEGGVLQQIEKDFGSFTNFREEFIRSALQLLGSGWVWLVLKRNERKLSVVHTRNAISPLAFGDIDDKLTYVTNFMDHLVSWHTVTLRMMRAESFVNLGEPNIPEA

>Zm00001d009990

MALRTLASKNALSFALGGAARPSAASARGVTTVALPDLSYDFGALEPAISGEIMRLHHQKHHATYVANYNKALEQLDAAVAKGDASAVVQLQGAIKFNGGGHVNHSIFWKNLKPISEGGGEPPHGKLGWAIDEDFGSFEALVKRMNAEGAALQGSGWVWLALDKEAKKLSVETTANQDPLVTKGASLVPLLGIDVWEHAYYLQWSPPLLSWLQYKNVRPDYLNNIWKVMNWKYAGEVYENVLA

>Zm00001d014632

MGYAEIAIRPSGSGKKIQRQGGLSRRFSNVSYYGLTTPPYKLDALEPYMSKRTVELHWGKHHQDYMDGLNNQLATSPLYGYTLEDLIKKAYNNDNPLPEYNNTAQHAYYLDYKDDKLTYVTNFMDHLVSWHTVTLRMMRAESFVNLGEPNILEA

>Zm00001d031908

MAAQSFLLAATAAARSPAVFAAPYSSARPFHSVHFVAGPWGAAAARALVVADATKKAVAVLKGASEVEGVVTLTQDDDGPTTVNVRITGLTPGLHGFHLHEFGDTTNGCISTGPHFNPNNLTHGAPEDEVRHAGDLGNIVANAEGIAEATIVDTQIPLTGPNSVVGRAFVVHELEDDLGKGGHELSLSTGNAGGRLACGVVGLTPL

>Zm00001d045384

MSKRTVELHWGKHHQDYVDGLNKQLATSPLYGYTLEDLIKEAYNNGNPLPEYNNAAQVWNHHFFWESMQPEGGGLPEGGVLQQIEKDFGSFTNFREEFIRSALQLLGSGWVWLVLKRNETKLSVVHTQNAIFPLAFGDIPIINLDLW

>Zm00001d045538

MASTALVAVAVGGAISLGLVASSGVASCSLRAGGDSRRFGPRLVLLRRGGAEGEGTRTRNCPIFRCANKVDVVTEDDSVDGDVTDDGEDLEDAADDAIDADVGTEDELESSLPEDVEWIKQQPLPYPLDALEPYISKETVEQHWGVHQQIHVDRLNGMIGGSEWEGMSLGQMMLASFNEGREQPHPPFFHAAQVWNHDFYWRSMKPGGGGKPPERLLKFINRDFGSHEGMIRQFMDAALTQFGSGWVWLSYKGSGLPYVKSRSPIPSDNHGRLVISKTPNAINPLVWGHSPLLAIDVWEHAYYLDYEDRRADYVSAILEKLVSWETVESRLAKAVARAVERDEHLRRRILRKQRLAQANGQSRARSRARQGRQGDQEVARSRPVEA
