# Supplementary figures and images for: Genome-wide identification and transcriptional expression analysis of superoxide dismutase (SOD) family in wheat (Triticum aestivum)
Source: PeerJ. 2019 Nov 19;7:e8062. doi: 10.7717/peerj.8062 (PMC6873880; doi:10.7717/peerj.8062)

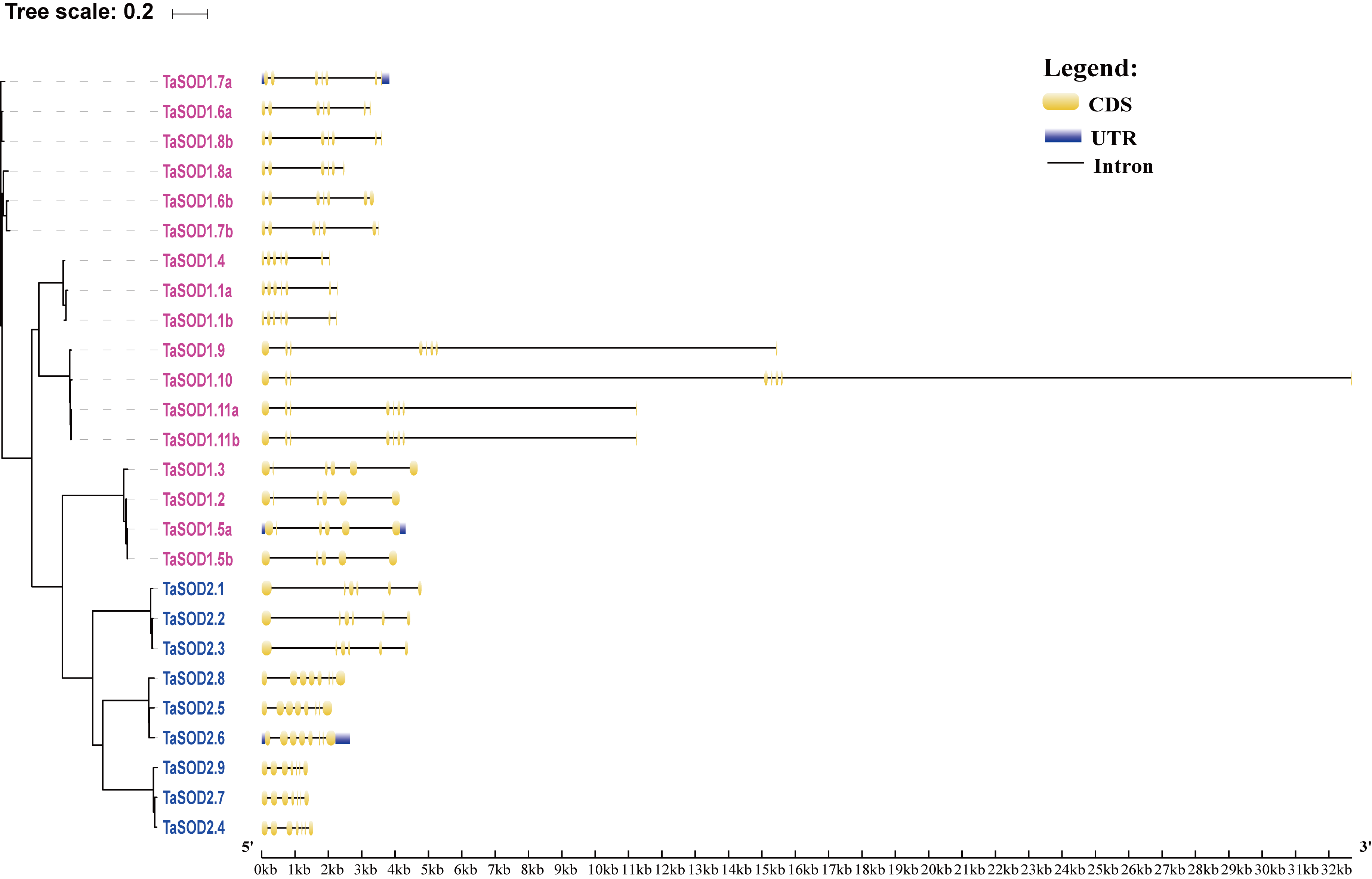

Supplement: Figure S1 [file peerj-07-8062-s007.png]

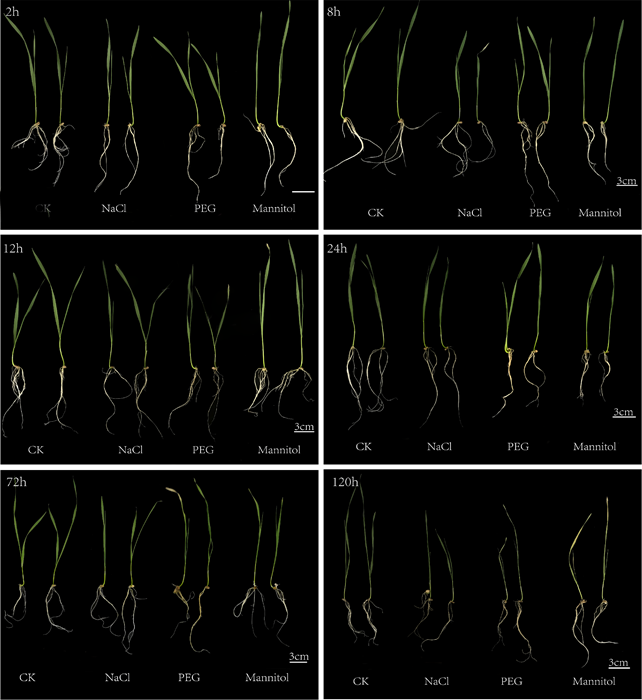

Supplement: Figure S2 [file peerj-07-8062-s008.png]
